# Predictors of HbA_1c_ treatment response to add-on medication following metformin monotherapy: a population-based cohort study

**DOI:** 10.1038/s41598-023-47896-x

**Published:** 2023-11-28

**Authors:** Wei Ying Tan, Wynne Hsu, Mong Li Lee, Ngiap Chuan Tan

**Affiliations:** 1 Saw Swee Hock School of Public Health, National University of Singapore, MD1 - Tahir Foundation Building, 12 Science Drive 2, #11, Singapore, 117549 Singapore; 2 Institute of Data Science, National University of Singapore, Singapore, Singapore; 3 School of Computing, National University of Singapore, Singapore, Singapore; 4 SingHealth Polyclinics, SingHealth, Singapore, Singapore; 5 Family Medicine Academic Clinical Programme, SingHealth-Duke NUS Academic Medical Centre, Singapore, Singapore

**Keywords:** Medical research, Metabolic disorders, Endocrine system and metabolic diseases

## Abstract

Evidence on the influence of patient characteristics on HbA_1c_ treatment response for add-on medications in patients with type 2 diabetes (T2D) is unclear. This study aims to investigate the predictors of HbA_1c_ treatment response for three add-on medications (sulfonylureas (SU), dipeptidyl peptidase-4 (DPP-4) and sodium–glucose cotransporter-2 (SGLT-2) inhibitor) in metformin monotherapy treated patients with T2D. This retrospective cohort study was conducted using the electronic health record data from six primary care clinics in Singapore. A total of 9748 adult patients with T2D on metformin monotherapy receiving SU, DPP-4 or SGLT-2 add-on were 1:1 propensity score matched to patients receiving other add-on medications. Patient demographics, laboratory results, diabetes related complications, comedications, and treatment response at two endpoints (HbA_1c_ reduction ≥ 1% at 6th month, HbA_1c_ goal attainment < 7% at 12th month) were examined. Multiple logistic regression analyses were used to identify patient characteristics associated with the treatment responses. After matching, there were 1073, 517, and 290 paired cohorts of SU, DPP-4 and SGLT-2 respectively. Besides baseline HbA_1c_, patients with longer hypertension disease duration and higher cholesterol HDL were associated with better treatment response to SU medication add-on. Lower estimated glomerular filtration rate (eGFR), and angiotensin-II receptor medications were associated with better treatment response to DPP-4 add-on. Lower cholesterol HDL, higher creatinine serum, absence of renal complications and beta-blockers medications were associated with better treatment response to SGLT-2 add-on. The cholesterol HDL, creatinine serum, eGFR, hypertension disease duration, angiotensin-II receptors and beta-blockers class of medications can influence the HbA_1c_ treatment response for SU, DPP-4 and SGLT-2 add-on medications. Knowing the patients’ characteristics that influence treatment response can assist in guiding clinical decisions when selecting the appropriate add-on medication, ultimately helping to prevent the development of diabetes-related complications.

## Introduction

Type 2 diabetes (T2D) is a progressive disease characterized by elevated levels of blood glucose and the need to gradually intensify therapy due to deficiency in insulin secretion and/or insulin resistance^1^. Patients with T2D are at high risk of multiple comorbidities, including obesity, hyperlipidaemia, hypertension and kidney disease^2^. Proper glycaemic control is essential to prevent vascular complications^3^. Metformin is the first-line therapy recommended to most patients given its long-term safety profile, availability, and low cost^4, 5^. However, metformin monotherapy can maintain an optimal glycaemic control only for a short term^6^. Previous studies suggest that as the disease progresses, metformin monotherapy may fail to control blood glucose due to increased insulin resistance and other metabolic events^7, 8^. Patients who fail to response to monotherapy often require an intensified treatment such as combination therapy to achieve adequate glycaemic control.

With newer oral medications, combinations of different medications may help to further improve glycaemic control^9^. Sulfonylureas (SU), dipeptidyl peptidase-4 inhibitors (DPP-4), and sodium glucose cotransporter 2 (SGLT-2) inhibitors are popular add-on medication options to metformin^10^. Each class of medications differs in their mechanisms of action, side effects, cost, risk and benefit profiles. For instance, DPP-4 have a lower risk of hypoglycaemia while SGLT-2 confers cardiorenal benefits and are associated weight loss^11, 12^.

For patients who require an add-on medication to metformin, the choice of add-on medication is often not obvious. The American and European guidelines on T2D management suggest tailoring the choice of the add-on medication based on individual demographics and clinical profile such as degree of hyperglycaemia, cardiovascular risk, renal function, and other comorbidities^13, 14^. While numerous clinical trials and observational studies have examined the efficacies of various antidiabetic medications in lowering HbA_1c_ levels alongside with weight gain, risk of hypoglycaemia and major adverse cardiovascular events, few studies have investigated how patient characteristics such as demographics, laboratory results, concomitant comorbidities, and comedications are associated with treatment response to add-on medications in a metformin therapy treated population^15–17^. Furthermore, clinical trials that examined patient characteristics associated with add-on medication treatment response were mostly conducted on Western population and/or otherwise, limited by the small study number. Treatment response to an add-on medication may be influenced by ethnicity with underlying factors such as genetic constitution, lifestyle and living environment. Hence, findings from these studies may not generalize to other populations due to biological and ethnic differences.

This study examines the predictors that determine patient response to three add-on medications (SU, DPP-4 and SGLT-2) in a metformin monotherapy multi-ethnic Asian population. Knowing the significant predictors that influence treatment response can assist in guiding clinical decisions when selecting the appropriate add-on medication, ultimately helping to prevent the development of diabetes-related complications.

## Methods

### Settings and study population

**Figure 1:**
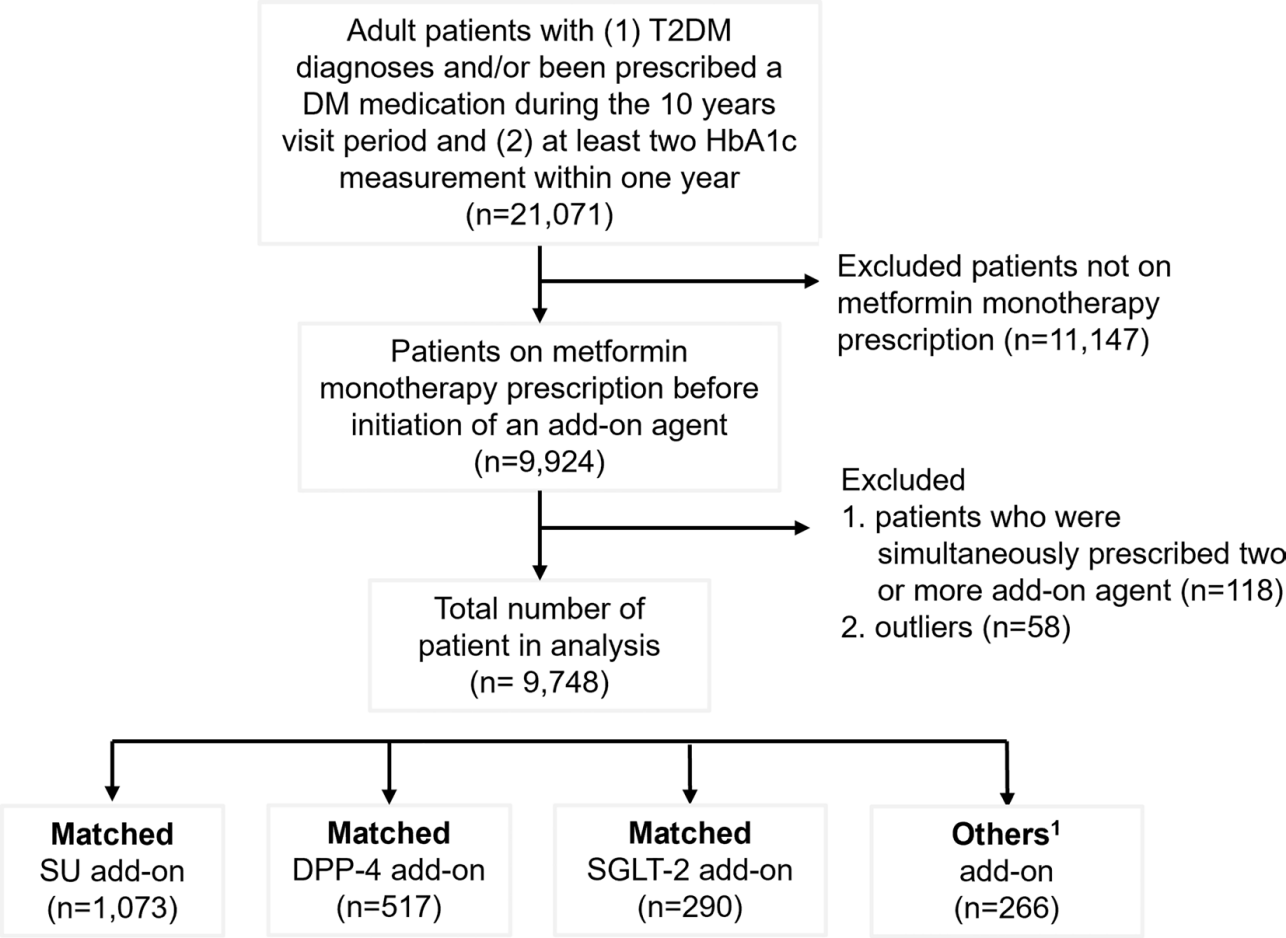
Flowchart of study population. ^1^Other refers to patients receiving alpha-glucosidase inhibitors or insulin add-on medications. Patients initiated with a medication add-on were matched 1:1 using a greedy nearest neighbour process without replacement, within a calliper distance of 0.2 SD of the logit of the propensity score to patients initiated with another type of add-on medication.

This retrospective cohort study was conducted using electronic health record (EHR) data obtained from six primary care clinics in Singapore. Ethics approval for this study was obtained from Institutional Review Board (IRB) of SingHealth (Reference Number: 2019/2604). The need for informed consent was waived by the ethics committee of SingHealth as the analysis was conducted on de-identified data. All methods were performed in accordance with relevant guidelines and regulations. As in previous study^18^, the study cohort comprises multi-ethnic Asians adult patients, aged ≥ 21 years, having T2D, hypertension (HTN) and/or hyperlipidaemia (HLD). Patient demographics, disease history, laboratory test results and medications prescriptions were extracted over a 10-year period from 1 January 2010 to 31 December 2019. Baseline was defined as the patient’s most recent EHR record prior to receiving an add-on medication for SU, DPP-4 and SGLT-2 after 1 January 2010. Patients were on metformin monotherapy if they were prescribed with metformin alone at baseline. Patients included in this study were (1) on metformin monotherapy at baseline, (2) have at least two HbA_1c_ measurements within one year, and (3) were initiated only one add-on medication of either SU, DPP-4 or SGLT-2. Patients (n = 58) with extreme values (beyond 4 standard deviations) in clinical measurements were excluded from analysis. In total, the study cohort consisted of 9748 patients. (See Fig. 1 Flowchart of study population).

### Patient variables

Demographic and clinical variables including age, sex, ethnicity, and BMI were obtained at baseline. Clinical variables consisted of blood pressure, comorbidities of HLD and HTN, duration of the three diseases diabetes, HTN and HLD, laboratory test results of HbA_1c,_ cholesterol, triglycerides (TG), creatinine serum, potassium serum and alanine transaminase (ALT) serum; and estimated glomerular filtration rate (eGFR) calculated using Chronic Kidney Disease Epidemiology Collaboration (CKD-EPI) creatinine equation^19^. We also included complications involving macrovascular, renal, eye and foot based on a pre-defined set ICD-10 codes, medications and patient referrals in Supplement Table S1 and Text S1. Finally, we considered the dose intensity of metformin and medications for HLD and HTN in our study. The total daily dose (TDS) of metformin is categorized into three intensity levels: low (L) for doses up to 1000 mg, moderate (M) for doses over 1000 mg but up to 2000 mg, and high (H) for doses exceeding 2000 mg. Medications for HLD and HTN were grouped by their classes. For example, losartan or valsartan belong to the medication class angiotensin-II-receptor. The list of medication classes can be found in Supplement Table S2.

### Outcome

The therapeutic efficacy of the add-on medications were evaluated based on two treatment responses of patients. The first response is whether they achieve a reduction in HbA_1c_ of at least 1% at the 6-month mark compared to baseline. The second response is whether they reach the HbA_1c_ goal of less than 7% at the 12-month mark. The 6-month HbA_1c_ reduction is considered a measure of short-term efficacy, while the 12-month HbA_1c_ attainment reflects long-term efficacy. Clinical investigation guidelines for T2D medicinal products recommend a minimum duration of 16 weeks for add-on medication to achieve its maximum effect and for HbA_1c_ levels to stabilize^20^. Clinical practice guideline on diabetes recommend HbA_1c_ testing every 6–12 months for patients with stable glycaemic control^21^. In addition to the two treatment responses, we have conducted an additional analysis using a more modest outcome: HbA_1c_ reduction of > 0.5% at the 6-month point. This supplementary criterion is to account for the differing potencies of various medications, thereby enhancing the comprehensiveness of our assessment on the shorter-term treatment response.

### Statistical analysis

To reduce data irregularities caused by variations in patients' visit schedules, such as delayed appointments or irregular testing, we employed interpolation techniques to generate monthly HbA_1c_ measurements^22^. In cases where a month had multiple HbA_1c_ values, the average of all values were used. The monthly data points provide a more consistent and granular dataset for analysis. Missing data on all other variables in the baseline EHR were imputed using the Multiple Imputation by Chained Equation (MICE)^23^. See Supplement Table S3 for the percentage of missing data in the variables. Propensity score analysis was conducted to balance covariates, including age, sex, ethnicity, BMI, diabetes duration, and baseline metformin TDS, between patient groups (i.e., SU add-on versus non-SU add-on, DPP-4 add-on versus non-DPP-4 add-on, and SGLT-2 add-on versus non-SGLT-2 add-on) at baseline. The propensity score was calculated as the predicted probability of receiving an add-on medication using a multivariable logistic regression model. The variables used for matching included age, gender, ethnicity, BMI, diabetes duration and baseline metformin TDS. Matching was performed for each type of add-on medications with the use of a 1:1 greedy nearest neighbor process without replacement, within a calliper distance of 0.2 SD of the logit of the propensity score^24^. The choice of 1:1 matching was made to enable a direct comparison of outcomes between the two groups (i.e., patients initiated with SU and non-SU medication add-on groups) and to provide straightforward and interpretable results^25^. Univariate analyses were used to identify variables associated with the two treatment outcomes. Variables that were found to be significant at *p*-value < 0.05 were selected for multivariable logistic regression analysis. All variables were normalized to a mean of 0 and a standard deviation of 1. Goodness of fit for all multivariable logistic regression model were evaluated using concordance statistic (C statistics). Multicollinearity among variables was assessed using Variance Inflation Factor (VIF). Variables with VIF > 10 were excluded from multivariate regression models. The odds ratios and 95% confidence interval (CI) for all variables on the two treatment outcomes were reported. All the analysis was performed using Python version 3.8.8, psmpy 0.3.13, tableone 0.7.12 and statmodel 0.12.2 libraries.

### Ethical approval

Ethics approval was obtained from SingHealth Centralized Institution Review Board (CIRB) in 2019 (SingHealth CIRB Reference: 2019/2604). Patient consent was not obtained as the analysis was conducted on de-identified data.

## Results

### Characteristics of the study population

Before matching, the average age of patients was 60.8 years (± 10.6), with 50.7% being male. Over a 10-year period, each patient had an average of 27.8 (± 9.4) HbA_1c_ measurements, with an average time interval of 3.9 months (± 2.2) between visits. At baseline, 88.6% of patients were initiated on a SU add-on, 5.3% on a DPP-4 add-on, and 3.0% on an SGLT-2 add-on. After matching, the analysis included 1,073 patients initiated with SU, 517 patients with DPP-4, and 290 patients with SGLT-2 add-on therapy. The baseline characteristics of the matched pairs are summarized in Table 1, and the distribution of propensity scores for the three add-on medications is illustrated in Supplement Fig. S1.

**Table 1:** Baseline characteristics of patients after matching.

	Sulfonylurea (SU) n = 1073	Other add-on	Dipeptidyl peptidase 4 inhibitors (DPP-4) n = 517	Other add-on	Sodium glucose co-transporter 2 inhibitors (SGLT-2) n = 290	Other add-on
Age	60.9 ± 10.5	60.4 ± 10.8	62.5 ± 10.7	62.3 ± 9.9	59.0 ± 9.0	58.7 ± 10.9
Sex (1 Male 0 Female)	482 (44.9)	496 (46.1)	216 (41.8)	221 (42.3)	129 (44.5)	116 (40.0)
Duration of metformin monotherapy prior to initiation of add-on medication (in years)	3.1 ± 1.9	3.0 ± 1.9	3.4 ± 1.8	3.2 ± 1.8	3.7 ± 1.8	3.6 ± 1.8
Time interval between visit (in months)	4.0 ± 4.0	3.9 ± 2.0	3.8 ± 1.0	3.8 ± 1.6	4.0 ± 1.5	3.8 ± 1.2
Race						
Chinese	732 (68.2)	750 (69.8)	353 (68.3)	346 (66.3)	199 (68.6)	187 (64.5)
Indian	126 (11.7)	123 (11.4)	66 (12.8)	72 (13.8)	36 (12.4)	44 (15.2)
Malay	152 (14.2)	170 (15.8)	64 (12.4)	86 (16.5)	41 (14.1)	45 (15.5)
Others	63 (5.9)	32 (3.0)	34 (6.6)	18 (3.4)	14 (4.8)	14 (4.8)
Body mass index (BMI)	27.4 ± 4.9	27.2 ± 4.7	26.8 ± 4.8	26.8 ± 4.5	28.7 ± 4.7	28.4 ± 5.1
Comorbidities						
Diabetes (DM)	14 (1.3)	26 (2.4)	8 (1.5)	7 (1.3)	50 (17.2)	34 (11.7)
Diabetes and hyperlipidaemia (DM and HLD)	153 (14.3)	135 (12.6)	76 (14.7)	69 (13.2)	226 (77.9)	244 (84.1)
Diabetes and hypertension (DM and HTN)	47 (4.4)	56 (5.2)	20 (3.9)	25 (4.8)	0	4 (1.4)
Diabetes hyperlipidaemia, hypertension (DHL)	859 (80.1)	858 (79.8)	413 (79.9)	421 (80.7)	14 (4.8)	8 (2.8)
Disease duration						
Diabetes (DM) years	3.2 ± 1.8	3.1 ± 1.7	3.3 ± 1.7	3.4 ± 1.7	3.8 ± 1.6	3.8 ± 1.7
Hyperlipidaemia (HLD) years	3.2 ± 1.9	2.9 ± 1.8	3.3 ± 1.8	3.1 ± 1.8	4.2 ± 1.7	3.5 ± 1.7
Hypertension (HTN) years	2.9 ± 2.1	2.7 ± 1.9	2.9 ± 2.0	3.0 ± 2.0	3.9 ± 2.2	3.2 ± 2.0
Lab test results						
Blood pressure systolic (SBP), mmHg	131.3 ± 15.3	130.1 ± 15.3	131.4 ± 15.5	130.7 ± 16.2	131.9 ± 13.8	131.2 ± 15.7
Blood pressure diastolic (DBP), mmHg	71.0 ± 9.0	71.6 ± 9.5	70.1 ± 8.8	70.8 ± 9.1	71.7 ± 8.9	71.4 ± 9.2
Glycated haemoglobin (HbA1c), % (continuous)	8.1 ± 1.2	8.1 ± 1.2	8.0 ± 1.0	8.1 ± 1.3	7.8 ± 0.9	8.1 ± 1.1
Cholesterol high-density lipoprotein (HDL), mmol/L	1.3 ± 0.3	1.3 ± 0.3	1.3 ± 0.3	1.3 ± 0.3	1.3 ± 0.3	1.3 ± 0.3
Cholesterol low-density lipoprotein (LDL), mmol/L	2.4 ± 0.6	2.4 ± 0.7	2.3 ± 0.6	2.4 ± 0.7	2.4 ± 0.6	2.3 ± 0.6
Total cholesterol, mmol/L	4.4 ± 0.7	4.4 ± 0.8	4.4 ± 0.7	4.4 ± 0.8	4.4 ± 0.7	4.3 ± 0.7
Triglycerides, mmol/L	1.6 [1.2,1.8]	1.6 [1.2,1.8]	1.6 [1.2,1.8]	1.5 [1.2,1.8]	1.6 [1.2,1.7]	1.6 [1.2,1.8]
Estimated glomerular filtration rate (eGFR)	87.9 ± 16.6)	88.5 ± 17.7)	86.7 ± 17.3)	88.2 ± 16.8)	93.3 ± 13.1	88.8 ± 19.0
Creatinine serum	72.1 ± 19.1	71.7 ± 19.4	72.5 ± 19.6	70.8 ± 20.2	67.2 ± 16.5	72.0 ± 20.4
Potassium serum	4.5 ± 0.4	4.4 ± 0.3	4.5 ± 0.4	4.5 ± 0.4	4.4 ± 0.3	4.5 ± 0.4
Alanine transaminase (ALT) serum	27.2 [20.0,31.9]	27.0 [18.0,32.0]	27.0 [19.0,31.2]	25.0 [17.0,30.6]	27.4 [21.0,33.1]	27.0 [17.2,33.0]
Metformin dose						
TDS ≤ 1000 mg	343 (32.0)	280 (26.0)	118 (22.8)	109 (20.9)	112 (38.6)	110 (37.9)
TDS 1000 mg to ≤ 2000 mg	422 (39.3)	528 (49.1)	272 (52.6)	270 (51.7)	121 (41.7)	144 (49.7)
TDS > 2000 mg	308 (28.7)	267 (24.8)	245 (47.4)	252 (48.3)	57 (19.7)	36 (12.4)
Number of HLD and HTN medications						
0 HLD medication	2 (0.2)	1 (0.1)	472 (91.3)	480 (92.0)	1 (0.3)	0
1 HLD medication	967 (90.1)	953 (88.7)	42 (8.1)	42 (8.0)	258 (89.0)	262 (90.3)
2 HLD medications	100 (9.3)	121 (11.3)	3 (0.6)	0	30 (10.3)	27 (9.3)
≥ 3 HLD medications	4 (0.4)	0	288 (55.7)	265 (50.8)	1 (0.3)	1 (0.3)
0 HTN medication	2 (0.2)	1 (0.1)	135 (26.1)	153 (29.3)	1 (0.3)	0
1 HTN medication	576 (53.7)	539 (50.1)	94 (18.2)	104 (19.9)	161 (55.5)	134 (46.2)
2 HTN medications	306 (28.5)	326 (30.3)	394 (76.2)	408 (78.2)	94 (32.4)	93 (32.1)
≥ 3 HTN medications	189 (17.6)	209 (19.4)	123 (23.8)	114 (21.8)	34 (11.7)	63 (21.7)
Existing HLD and HTN medications						
HMG-CoA reductase inhibitors (statins)	947 (88.3)	929 (86.4)	48 (9.3)	49 (9.4)	256 (88.3)	261 (90.0)
Fibric acid derivatives (gemfibrozil, fenofibrate)	113 (10.5)	141 (13.1)	12 (2.3)	1 (0.2)	29 (10.0)	32 (11.0)
Other lipid-lowering medications (ezetimibe, cholestyramine)	18 (1.7)	3 (0.3)	45 (8.7)	85 (16.3)	6 (2.1)	1 (0.3)
Diuretics	108 (10.1)	140 (13.0)	161 (31.1)	164 (31.4)	24 (8.3)	38 (13.1)
Beta blockers	332 (30.9)	330 (30.7)	104 (20.1)	152 (29.1)	86 (29.7)	91 (31.4)
ACE inhibitors	276 (25.7)	355 (33.0)	235 (45.5)	179 (34.3)	51 (17.6)	91 (31.4)
Angiotensin-II receptor	419 (39.0)	368 (34.2)	210 (40.6)	227 (43.5)	113 (39.0)	112 (38.6)
Calcium antagonists	449 (41.8)	482 (44.8)	4 (0.8)	7 (1.3)	117 (40.3)	130 (44.8)
Other BP-lowering medications (Alpha blockers, direct vasodilators, sympatholytic)	7 (0.7)	10 (0.9)	0	0	1 (0.3)	10 (3.4)
Complications						
Macrovascular	245 (22.8)	219 (20.4)	118 (22.8)	105 (20.1)	54 (18.6)	55 (19.0)
Renal	217 (20.2)	236 (22.0)	201 (38.9)	202 (38.7)	23 (7.9)	75 (25.9)
Eye	425 (39.6)	378 (35.2)	16 (3.1)	24 (4.6)	126 (43.4)	104 (35.9)
Foot	42 (3.9)	41 (3.8)	221 (42.7)	247 (47.3)	9 (3.1)	12 (4.1)

Continuous variables with normal distribution have the format means ± SDs, while continuous variables with nonnormal distribution have the format medians [lower quartile, upper quartile]. Categorical variables were presented in counts (n) and percentages. SU: Sulfonylureas; DPP-4 inhibitor: Dipeptidyl peptidase-4 inhibitor; SGLT-2 inhibitor: Sodium–glucose cotransporter-2 inhibitor.

Patients who received SU add-on medication had a mean age of 60.9 (± 10.5) years, with 44.9% being male. In the matched cohort, patients had longer diabetes disease duration compared to the unmatched cohort. Among those who received SU add-on, 88.3% were on HMG-CoA reductase inhibitors class of medications and over a third had eye complications. Patients who received DPP-4 add-on were slightly older, have a lower BMI and over 40% of them were on  ACE inhibitors and angiotensin-II receptor class of medications. 42.7% and 38.9% of patients who received DPP-4 add-on also have foot and renal complications. Patients who received SGLT-2 add-on have lower baseline HbA_1c_, better renal function with lower creatinine serum, higher eGFR and fewer renal complications at baseline compared to patients who received other add-on medication. Similar to those on SU add-on medication, over 80% of patients who received SGLT-2 add-on were on HMG-CoA reductase inhibitors class of medications and around 40% were on angiotensin-II receptor and calcium antagonists’ class of medications.

### Univariate analysis

In the univariate analysis, for SU add-on, the baseline HbA_1c_ and disease duration of HTN were found to be significant factors for both HbA_1c_ reduction and HbA_1c_ goal attainment. Additionally, variables such as age, disease duration of DM and HLD, cholesterol HDL, LDL and TG, eGFR, creatinine, potassium and ALT serum, number of HTN medications, and calcium antagonist medications class were associated with HbA_1c_ goal attainment but not with HbA_1c_ reduction. In the case of DPP-4 add-on medication, most variables appeared insignificant except baseline HbA_1c_, cholesterol LDL and number of HLD medications. Variables such as age, BMI, disease duration of HLD and HTN, creatinine serum, eGFR, number of HTN medications, and HTN medication class of angiotensin-II receptor and other lipid-lowering medications (i.e. ezetimibe, cholestyramine) were associated with HbA_1c_ goal attainment but not with HbA_1c_ reduction. For SGLT-2 add-on medication, baseline HbA_1c_, diastolic blood pressure and cholesterol HDL were associated with HbA_1c_ reduction. While baseline HbA_1c_, creatinine serum, existing renal complications, and HTN medications of beta blockers were associated with HbA_1c_ goal attainment. All results of the univariate analysis can be found in Supplement Table S5–S7. The findings on the supplementary criterion HbA_1c_ reduction of > 0.5% at 6-month point were detailed in Supplemental materials—Text S2 and Fig. S2.

### Multivariate analysis

**Figure 2:**
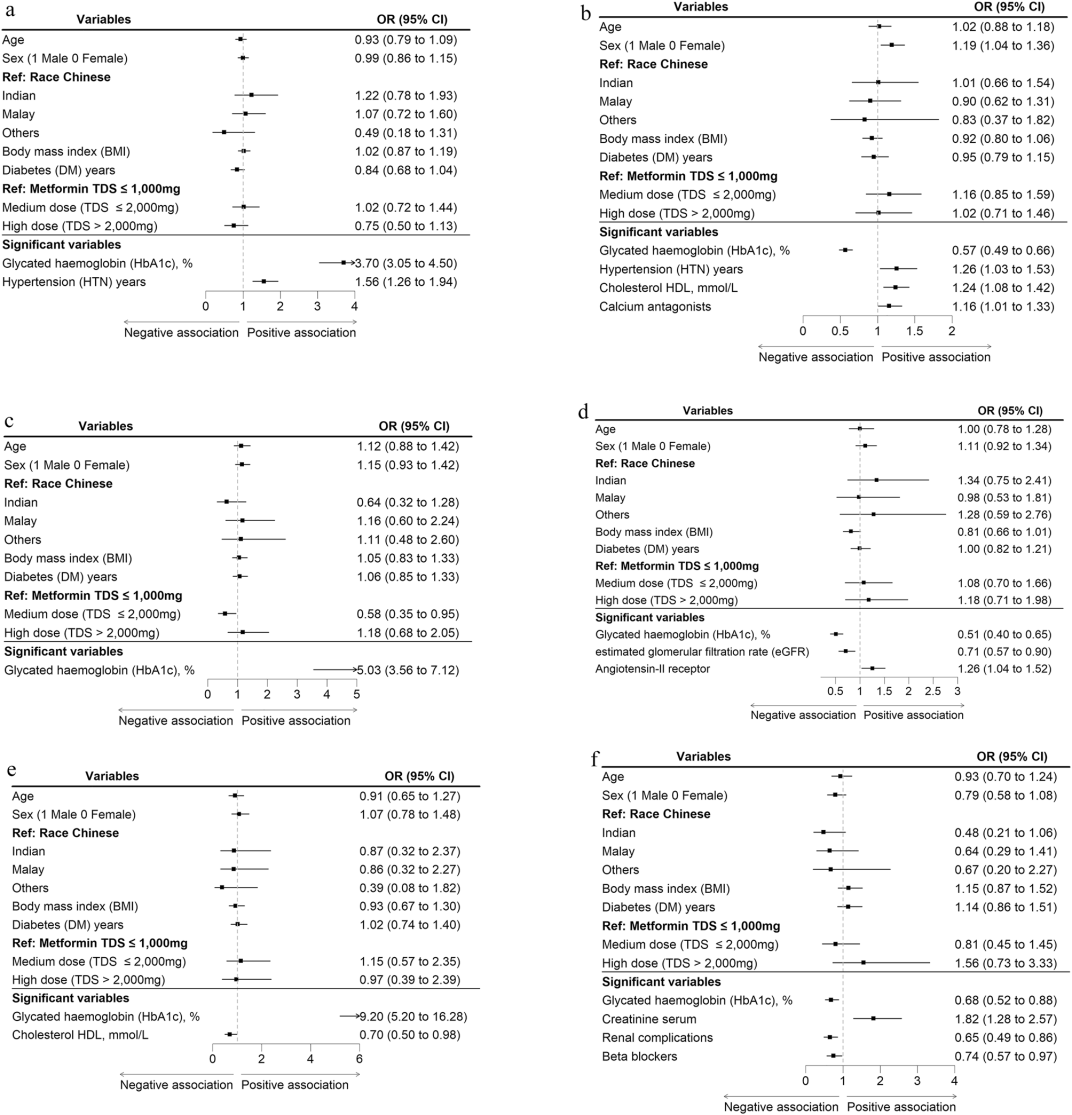
Patient variables associated with treatment response for add-on medication. (**A**) For SU add-on, HbA_1c_ reduction ≥ 1% measured at 6th month. (**B**) For SU add-on, HbA_1c_ goal attainment < 7% measured at 12th month. (**C**) For DPP-4 add-on, HbA_1c_ reduction ≥ 1% measured at 6th month. (**D**) For DPP-4 add-on, HbA_1c_ goal attainment < 7% measured at 12th month. (**E**) For SGLT-2 add-on, HbA_1c_ reduction ≥ 1% measured at 6th month. (**F**) For SGLT-2 add-on, HbA_1c_ goal attainment < 7% measured at 12th month. Column in right indicates odds ratio and its 95% confidence interval (CI) in brackets. Black box represents the odds ratio, and the horizontal line represents the 95%CI. Dotted vertical indicates line of null effect. All models were adjusted for age, sex, race, BMI, disease duration of diabetes and baseline metformin total daily dose.

In the multivariate analysis, patient variables associated with treatment response for SU add-on are shown in Fig. 2A,B. Higher HbA_1c_ and longer HTN disease duration were independently associated with increased odds of HbA_1c_ reduction. Lower baseline HbA_1c_, longer HTN duration, higher cholesterol HDL and HTN medication class calcium antagonists were independently associated with increased odds of HbA_1c_ goal attainment. The effect estimates and 95%CI for all variables at the 6th and 12th months can be found in Supplement Tables S8–S9. The C-statistics for HbA_1c_ reduction and HbA_1c_ goal attainment are provided in Supplement Fig. S3A,B. Figure 2C,D shows the patient variables associated with treatment response for DPP-4 add-on. Only higher baseline HbA_1c_ were associated with increased odds of HbA_1c_ reduction; while patients with lower baseline HbA_1c_, lower eGFR and having HTN medication class angiotensin-II receptor were more likely to achieve HbA_1c_ goal attainment at 12th month.

Lastly, Fig. 2E, Fshows the patient variables associated with treatment response for SGLT-2 add-on. Higher HbA_1c_, lower cholesterol HDL at baseline were associated with increased odds of HbA_1c_ reduction and higher creatinine serum, absence of renal complications and HTN medication class of beta blockers were associated with increased odds of HbA_1c_ goal attainment.

## Discussion

In this retrospective study of metformin monotherapy treated Asian population, baseline HbA_1c_, HTN disease duration, cholesterol HDL, creatinine serum, eGFR, existing renal complications, and HTN medication class of calcium antagonists, angiotensin-II receptor and beta blockers were independently associated with HbA_1c_ outcome and were predictive of treatment response for SU, DPP-4 and SGLT-2 add-on medications.

Consistent with the previous studies^26–28^, our regression models demonstrated that baseline HbA_1c_ was a significant predictor of treatment response for all three add-on medications. Patients with higher baseline HbA_1c_ were more likely to achieve a reduction of ≥ 1% in HbA_1c_ levels at the 6th-month mark after initiating add-on medication. These findings aligned with studies investigating the use of SU, DPP-4 and SGLT-2 as add-on for patients on metformin monotherapy^17, 27^. In our study, baseline HbA_1c_ were observed to be inversely associated with HbA_1c_ goal attainment. This suggest that patients with higher baseline HbA_1c_ were less likely to achieve HbA_1c_ of < 7% at 12th month after second medication was added.

BMI showed a moderate association (*p*-value < 0.10) with HbA_1c_ treatment response for SU and DPP-4 add-on medications in the univariate analysis. When considering other factors, BMI maintained a moderate association (*p*-value < 0.08) with HbA_1c_ goal attainment specifically for DPP-4 add-on medications. The finding suggests that for every unit of decrease in BMI, there was approximately 96% (1/odd ratio of 0.51) increase in odds of patient achieving HbA_1c_ goal attainment. Our finding on BMI concurred with the TriMaster clinical trial on DPP-4 and SGLT-2 as second- or third-line therapy among T2D patients on metformin monotherapy or metformin and SU therapy. The study found patients with BMI ≤ 30 kg/m^2^ achieved greater reduction in HbA_1c_ when treated with DPP-4 add-on medication compared to those with BMI > 30 kg/m^2^^29^.

For DPP-4 add-on, we observed that patients with lower eGFR had a higher likelihood of achieving HbA_1c_ goal attainment. Limited studies have compared HbA_1c_ treatment response among patients with varying degrees of renal impairment. However, a study conducted on 1101 Asian patients with T2D and mild impaired renal function concluded that DPP-4, when used alone or in combination with other glucose-lowering medications, provides protection against the decline in renal function^30^. This finding was further supported by a clinical review that suggest DPP-4 medications were safe and well tolerated in T2D patients with renal impairment^11^.

For SGLT-2 add-on, eGFR was not significantly associated with treatment response in our multivariable model. Instead, we found patients without renal complications at baseline were more likely to achieve an HbA_1c_ goal attainment of < 7%, compared to patients with existing renal complications. The reduced glucose filtration and resulting modest glycosuria with SGLT-2 therapy among patients with impaired renal function may explain the attenuated glucose-lowering efficacy compared to patients without established renal impairment^31, 32^. The TriMaster study supported our findings, showing that lower eGFR was associated with a reduced glucose-lowering response to SGLT-2 inhibitors^29^. Nevertheless, previous clinical studies have provided evidence that SGLT-2 inhibitors can delay the progression of chronic kidney disease (CKD) and can be administered to patients with heart failure or CKD, or at risk for adverse cardiac or renal pathologies^12^. In this study, we observed that patients with higher serum creatinine levels were more likely to achieve their HbA_1c_ goal than patients with lower serum creatinine levels at baseline. Preliminary findings showed that patients initiated with SGLT-2 add-on medications had lower baseline serum creatinine levels (67.2 ± 16.5) compared to patients using other add-on medications, such as SU (72.8 ± 18.3) and DPP-4 (72.5 ± 19.6). Hence, we caution that the findings related to serum creatinine may not be generalized beyond the range defined by the characteristics of the patients in this dataset.

In our multivariable models, variables such as cholesterol HDL and disease duration of HTN were predictive of HbA_1c_ treatment response. However, previous studies have shown mixed results regarding the predictive value of these variables. A systematic review^33^ on the clinical predictors of treatment response to metformin and SU found that blood pressure, cholesterol, triglycerides, and macrovascular complications influenced the response to add-on medications; whereas the study conducted on an Asian population with T2D^34^ concluded that blood pressure, cholesterol, and comorbidities were not significant predictors. We reasoned that these discrepancies may be attributed the underlying differences in the sample size, study population, medication initiated and other factors such as diet and lifestyle.

The current study also found potential associations between several HTN medication classes, such as beta blockers, calcium antagonists and angiotensin-II receptor blockers, and HbA_1c_ treatment response, indicating a possible link between HTN medications and HbA_1c_ response. Another study suggested that certain HTN medication classes, like channel blockers, provide protection against hypoglycaemia when used with insulin secretagogues like SU^35^. However, further detailed analysis is required to better understand the mechanism and establish a more conclusive link between HTN medications and add-on medication treatment response.

The study has several strengths that contribute to its robustness and generalizability. Firstly, this study draws data from primary care clinics in Singapore that serve large and diverse patient populations. Patients comprise of individuals representative of the community, including individuals of different ages, ethnic groups, socioeconomic backgrounds, and health conditions. Secondly, the use of data from multiple clinics also reduces the risk of selection bias, which can occur when data is collected from a single site. Thirdly, the large sample size enabled the examination of various factors such as demographics, laboratory results, comorbidities, and medication classes related to HLD and HTN and their impact on HbA_1c_ treatment response.

This study also has several limitations that should be considered. First, majority of T2D patients on metformin monotherapy who visited the polyclinic during the period 2010–2019 were prescribed SU add-on medication. This was because the local committee’s recommendations for dual therapy with DPP-4 and SGLT-2 inhibitors alongside metformin were introduced relatively recently in 2016 and reaffirmed in 2020. Hence, changes in prescription practice will take time to be reflected in EHR records. Second, the list of medications analysed was limited to those included on the institution's approved drug list. Specific medications, such as glibenclamide under SU, were absent in our analysis due to recommendations by the local health authority regarding its use, which is associated with a higher risk of hypoglycaemia compared to other SUs. Third, the choice and dosage of the add-on medication in the EHR were subjected to clinician's judgment, the cost of medications, and the patient's preferences, rather than being based solely on clinical outcomes. To minimize potential bias from baseline differences, propensity score matching was employed in our study. Other factors, such as demographics and the total daily dosage of metformin at baseline that could affect glycaemic response, were controlled by adding potential confounding variables in multivariable models. Fourth, bias may have been introduced by excluding patients with fewer than two HbA_1c_ measurements within one year and those who were prescribed two or more add-on medications concurrently, potentially affecting the generalizability of our study findings. Fifth, our study focused on several a priori known risk factors for T2D and its complications. Additional factors such as diet, lifestyle, genetic risk factors, medication contraindications, and compliance that might influence add-on medication treatment response were not accounted for in the present study. Finally, the polyclinic patients primarily consist of individuals without or with limited private health insurance coverage, as they offer more affordable healthcare options compared to specialized/private facilities. Therefore, the findings of this study may not fully generalize to patients attending specialized facilities. Despite these limitations, this study incorporated real-world data and provides clinicians with evidence to select the optimal choice of add-on medication for better glycaemic control.

### Implications for research and practice

Findings from this study can potentially be used to support clinicians in selecting the optimal add-on medication and avoid development of diabetes related complications. A future avenue for our study is to develop a system that recommends an optimal add-on medication following metformin monotherapy. It will be integrated into our existing PERsonalized DIabetes Counselling Tool using Artificial Intelligence to support clinical decision-making in a multi-site trial^22, 36^.

## Conclusions

In this multi-ethnic Asian population with T2D treated with metformin therapy, longer HTN disease duration, lower cholesterol HDL and calcium antagonists medication class were associated with better treatment response to SU medication add-on. Lower eGFR and angiotensin-II receptor medications were associated with better treatment response to DPP-4 add-on. Lower cholesterol HDL, higher creatinine serum, absence of renal complications and beta-blockers medications were associated with better treatment response to SGLT-2 add-on. These findings highlight the importance of considering cholesterol profile, renal function, presence of hypertension comorbidity, and comedications when selecting add-on medications.

### Supplementary Information

Supplementary Information.

## Data Availability

The datasets analysed in the current study are not publicly available as they contain information that are sensitive to the institution. They may be made available from the corresponding author on reasonable request.

## Abbreviations

AG: Alpha-glucosidase inhibitors
ALT: Alanine transaminase
BMI: Body mass index
CPG: Clinical practice guidelines
DPP-4 inhibitor: Dipeptidyl peptidase-4 inhibitor
DHL: Diabetes, hyperlipidaemia and hypertension
EHR: Electronic health records
eGFR: Estimated glomerular filtration rate
HbA_1c_: Glycated haemoglobin
HDL: Cholesterol high-density lipoprotein
HLD: Hyperlipidaemia
HTN: Hypertension
ICD: International classification of diseases
LDL: Cholesterol high-density lipoprotein
SGLT-2 inhibitor: Sodium–glucose cotransporter-2 inhibitor
SU: Sulfonylureas
T2D: Type 2 diabetes
TDS: Total daily dose
