# Supplementary material for: Predictors of HbA1c treatment response to add-on medication following metformin monotherapy: a population-based cohort study
Source: Sci Rep. 2023 Nov 28;13:20891. doi: 10.1038/s41598-023-47896-x (PMC10684587; doi:10.1038/s41598-023-47896-x)
Supplement: Supplementary file 1 — Supplementary Information. [file 41598_2023_47896_MOESM1_ESM.docx]

## **Predictors of HbA1c treatment response to add-on medication following metformin monotherapy: A Population-Based Cohort Study**

**Supplemental Data**

**Table S1** ICD-10 codes related complications of macrovascular, renal, eye and foot

| **Complications** | **ICD-10 codes** |
| --- | --- |
| Eye | E1431, 3620 |
| Foot | E1140, E1473, I739, 4439 |
| Renal | E1122, 25040, N183, N184, N185, 5859, 585 |
| Macrovascular | I249, I259, 4149, I500, 4280, G459, I64, 4349 |

Abbreviation: ICD-10 = International Classification of Diseases, 10^th^ Revision.

**Text S1** Identification of additional cases related complications of macrovascular, renal, eye and foot

Complications involving macrovascular, renal, eye and foot based on a pre-defined set ICD-10 codes, medications and patient referrals in Supplement Table S1. In addition, patients were considered to have macrovascular complication if they had been prescribed the following antiplatelet medications: aspirin, clopidogrel, dipyridamole or ticagrelor; renal complication if they had eGFR <60ml/min/1.73m^2^; eye complication if they had a diabetic referable finding on eye examination and/or were on follow-up with an eye specialist; and foot complication if they have been flagged as high risk for foot ulcer during an examination and/or were on follow-up with a podiatrist.

**Table S2** Medication type and classes

| **Medication class** | | **Medications type** |
| --- | --- | --- |
| Diabetes medication | Biguanides | Metformin |
|  | Sulfonylureas | Gliclazide |
|  |  | Glipizide |
|  |  | Tolbutamide |
|  | DPP-4 inhibitors | Linagliptin |
|  |  | Sitagliptin |
|  |  | Vildagliptin |
|  | SGLT-2 inhibitors | Dapagliflozin |
|  |  | Empagliflozin |
|  | Insulin^1^ | Actrapid |
|  |  | Apidra |
|  |  | Insulatard |
|  |  | Lantus |
|  |  | Levemir |
|  |  | Mixtard |
|  |  | Novomix |
|  |  | Novorapid |
| Lipid-lowering medications | HMG-CoA reductase inhibitors | Atorvastatin |
|  |  | Lovastatin |
|  |  | Pravastatin |
|  |  | Rosuvastatin |
|  |  | Simvastatin |
|  | Fibric acid derivatives | Gemfibrozil |
|  |  | Fenofibrate |
|  | Other lipid-lowering agents | Ezetimibe |
|  |  | Cholestyramine |
| Anti-hypertensive medications | Diuretics | Hydrochlorothiazide |
|  |  | Hydrochlorothiazide Amiloride |
|  |  | Indapamide |
|  |  | Spironolactone |
|  | Beta blockers | Atenolol |
|  |  | Bisoprolol |
|  |  | Nifetex |
|  |  | Propranolol |
|  | ACE inhibitors | Captopril |
|  |  | Enalapril |
|  |  | Lisinopril |
|  |  | Perindopril |
|  | Angiotensin-II receptor | Candesartan |
|  |  | Losartan |
|  |  | Olmesartan |
|  |  | Telmisartan |
|  |  | Valsartan |
|  | Calcium antagonists | Amlodipine |
|  |  | Nifetex |
|  |  | Nifedipinela |
|  | Other BP-lowering agents | Prazosin |
|  |  | Methyldopa |
|  |  | Hydralazine |

**Table S3** Missing data by variables

| Variables | Count | Pct |
| --- | --- | --- |
| Body mass index (BMI) | 405 | 2.51 |
| Blood pressure systolic (SBP), mmHg | 9 | 0.06 |
| Blood pressure diastolic (DBP), mmHg | 9 | 0.06 |
| Cholesterol high-density lipoprotein (HDL), mmol/L | 4,436 | 27.49 |
| Cholesterol low-density lipoprotein (LDL), mmol/L | 4,481 | 27.77 |
| Total cholesterol, mmol/L | 4,432 | 27.46 |
| Triglycerides, mmol/L | 4,439 | 27.51 |
| ALT serum | 4,377 | 27.12 |
| Creatinine serum | 4,416 | 27.36 |
| ALT serum^1^ | 4,304 | 26.67 |

**Table S4** Baseline characteristics before matching

|  | **Overall n=9,748** | **Sulfonylurea (SU) n=8,675** | **Dipeptidyl peptidase 4  inhibitors (DPP4) n=517** | **Sodium glucose  co-transporter 2 inhibitors (SGLT2) n=290** | **Others n=266** |
| --- | --- | --- | --- | --- | --- |
| Age | 60.8 ± 10.6 | 4462 ± 51.4 | 216 ± 41.8 | 129 ± 44.5 | 137 ± 51.5 |
| Sex (1 Male 0 Female) | 4944 (50.7) | 2.1 (1.6) | 3.4 (1.8) | 3.7 (1.8) | 1.8 (1.7) |
| Duration of metformin monotherapy prior to initiation of add-on medication (in years) | 2.2 ± 1.7 | 3.9 ± 1.9 | 3.8 ± 1.0 | 4.0 ± 1.5 | 4.6 ± 7.8 |
| Time interval between visit (in months) | 3.9 ± 2.2 | 3.9 ± 1.9 | 3.8 ± 1.0 | 4.0 ± 1.5 | 4.6 ± 7.8 |
| **Race** |  |  |  |  |  |
| Chinese | 6617 (67.9) | 5885 (67.8) | 353 (68.3) | 199 (68.6) | 180 (67.7) |
| Indian | 1031 (10.6) | 905 (10.4) | 66 (12.8) | 36 (12.4) | 24 (9.0) |
| Malay | 1677 (17.2) | 1525 (17.6) | 64 (12.4) | 41 (14.1) | 47 (17.7) |
| Others | 423 (4.3) | 360 (4.1) | 34 (6.6) | 14 (4.8) | 15 (5.6) |
| Body mass index (BMI) | 27.2 ± 4.8 | 27.2 ± 4.8 | 26.8 ± 4.8 | 28.7 ± 4.7 | 27.0 ± 5.0 |
| **Comorbidities** |  |  |  |  |  |
| Diabetes (DM) | 231 (2.4) | 217 (2.5) | 8 (1.5) | 0 | 6 (2.3) |
| Diabetes and hyperlipidaemia (DM and HLD) | 1379 (14.1) | 1226 (14.1) | 76 (14.7) | 50 (17.2) | 27 (10.2) |
| Diabetes and hypertension (DM and HTN) | 519 (5.3) | 472 (5.4) | 20 (3.9) | 14 (4.8) | 13 (4.9) |
| Diabetes hyperlipidaemia, hypertension (DHL) | 7619 (78.2) | 6760 (77.9) | 413 (79.9) | 226 (77.9) | 220 (82.7) |
| **Disease duration** |  |  |  |  |  |
| Diabetes (DM) years | 2.3 ± 1.5 | 2.2 ± 1.4 | 3.3 ± 1.7 | 3.8 ± 1.6 | 2.1 ± 1.6 |
| Hyperlipidaemia (HLD) years | 2.3 ± 1.6 | 2.1 ± 1.6 | 3.3 ± 1.8 | 4.2 ± 1.7 | 1.8 ± 1.5 |
| Hypertension (HTN) years | 2.1 ± 1.7 | 2.0 ± 1.6 | 2.9 ± 2.0 | 3.9 ± 2.2 | 1.7 ± 1.6 |
| **Lab test results** |  |  |  |  |  |
| Blood pressure systolic (SBP), mmHg | 131.3 ± 16.0 | 131.3 ± 16.1 | 131.4 ± 15.5 | 131.9 ± 13.8 | 130.5 ± 16.6 |
| Blood pressure diastolic (DBP), mmHg | 71.8 ± 9.5 | 71.9 ± 9.6 | 70.1 ± 8.8 | 71.7 ± 8.9 | 71.9 ± 9.3 |
| Glycated haemoglobin (HbA_1c_), % | 8.2 ± 1.3 | 8.2 ± 1.3 | 8.0 ± 1.0 | 7.8 ± 0.9 | 8.5 ± 1.8 |
| Cholesterol high-density lipoprotein (HDL), mmol/L | 1.3 ± 0.3 | 1.3 ± 0.3 | 1.3 ± 0.3 | 1.3 ± 0.3 | 1.3 ± 0.2 |
| Cholesterol low-density lipoprotein (LDL), mmol/L | 2.4 ± 0.7 | 2.4 ± 0.7 | 2.3 ± 0.6 | 2.4 ± 0.6 | 2.5 ± 0.7 |
| Total cholesterol, mmol/L | 4.5 ± 0.7 | 4.5 ± 0.8 | 4.4 ± 0.7 | 4.4 ± 0.7 | 4.5 ± 0.8 |
| Triglycerides, mmol/L | 1.6 [1.3,1.8] | 1.6 [1.3,1.8] | 1.6 [1.2,1.8] | 1.6 [1.2,1.7] | 1.6 [1.4,1.7] |
| estimated glomerular filtration rate (eGFR) | 88.0 ± 16.3 | 88.0 ± 16.2 | 86.7 ± 17.3 | 93.3 ± 13.1 | 84.3 ± 17.4 |
| Creatinine serum | 72.7 ± 18.4 | 72.8 ± 18.3 | 72.5 ± 19.6 | 67.2 ± 16.5 | 76.9 ± 19.5 |
| Potassium serum | 4.4 ± 0.3 | 4.4 ± 0.3 | 4.5 ± 0.4 | 4.4 ± 0.3 | 4.5 ± 0.4 |
| Alanine transaminase (ALT) serum | 27.4 [20.0,31.8] | 27.4 [20.0,31.8] | 27.0 [19.0,31.2] | 27.4 [21.0,33.1] | 27.4 [19.2,31.2] |
| **Metformin dose** |  |  |  |  |  |
| TDS ≤ 1,000mg | 3336 (34.2) | 2993 (34.5) | 178 (34.4) | 112 (38.6) | 53 (19.9) |
| TDS 1,000mg to ≤ 2,000mg | 4313 (44.2) | 3891 (44.9) | 221 (42.7) | 121 (41.7) | 80 (30.1) |
| TDS > 2,000mg | 2099 (21.5) | 1791 (20.6) | 118 (22.8) | 57 (19.7) | 133 (50.0) |
| **Number of HLD and HTN medications** |  |  |  |  |  |
| 0 HLD medication | 19 (0.2) | 17 (0.2) | 0 | 1 (0.3) | 1 (0.4) |
| 1 HLD medication | 8826 (90.5) | 7859 (90.6) | 472 (91.3) | 258 (89.0) | 237 (89.1) |
| 2 HLD medications | 895 (9.2) | 795 (9.2) | 42 (8.1) | 30 (10.3) | 28 (10.5) |
| ≥ 3 HLD medications | 8 (0.1) | 4 (0.0) | 3 (0.6) | 1 (0.3) | 0 |
| 0 HTN medication | 19 (0.2) | 17 (0.2) | 0 | 1 (0.3) | 1 (0.4) |
| 1 HTN medication | 4924 (50.5) | 4348 (50.1) | 288 (55.7) | 161 (55.5) | 127 (47.7) |
| 2 HTN medications | 2889 (29.6) | 2583 (29.8) | 135 (26.1) | 94 (32.4) | 77 (28.9) |
| ≥ 3 HTN medications | 1916 (19.7) | 1727 (19.9) | 94 (18.2) | 34 (11.7) | 61 (22.9) |
| **Existing HLD and HTN medications** |  |  |  |  |  |
| HMG-CoA reductase inhibitors (statins) | 8504 (87.2) | 7557 (87.1) | 457 (88.4) | 256 (88.3) | 234 (88.0) |
| Fibric acid derivatives (gemfibrozil, fenofibrate) | 1070 (11.0) | 957 (11.0) | 48 (9.3) | 29 (10.0) | 36 (13.5) |
| Other lipid-lowering medications (ezetimibe, cholestyramine) | 53 (0.5) | 35 (0.4) | 12 (2.3) | 6 (2.1) | 0 |
| Diuretics | 1265 (13.0) | 1157 (13.3) | 45 (8.7) | 24 (8.3) | 39 (14.7) |
| Beta blockers | 3041 (31.2) | 2709 (31.2) | 161 (31.1) | 86 (29.7) | 85 (32.0) |
| ACE inhibitors | 3125 (32.1) | 2849 (32.8) | 104 (20.1) | 51 (17.6) | 121 (45.5) |
| Angiotensin-II receptor | 3237 (33.2) | 2818 (32.5) | 235 (45.5) | 113 (39.0) | 71 (26.7) |
| Calcium antagonists | 4342 (44.5) | 3893 (44.9) | 210 (40.6) | 117 (40.3) | 122 (45.9) |
| Other BP-lowering medications (Alpha blockers, direct vasodilators, sympatholytics) | 116 (1.2) | 109 (1.3) | 4 (0.8) | 1 (0.3) | 2 (0.8) |
| **Complications** |  |  |  |  |  |
| Macrovascular | 2089 (21.4) | 1844 (21.3) | 123 (23.8) | 54 (18.6) | 68 (25.6) |
| Renal | 1741 (17.9) | 1524 (17.6) | 118 (22.8) | 23 (7.9) | 76 (28.6) |
| Eye | 3327 (34.1) | 2902 (33.5) | 201 (38.9) | 126 (43.4) | 98 (36.8) |
| Foot | 366 (3.8) | 324 (3.7) | 16 (3.1) | 9 (3.1) | 17 (6.4) |

Continuous variables with normal distribution have the format means ± SDs, while continuous variables with nonnormal distribution has the format medians [lower quartile, upper quartile]. Categorical variables were presented in counts (n) and percentages. SU: Sulfonylureas; DPP-4 inhibitor: Dipeptidyl peptidase-4 inhibitor; SGLT-2 inhibitor: Sodium-glucose cotransporter-2 inhibitor.

**Figure S1** Box-and-whiskers plot of the distribution of the propensity scores in the matched

| **(A)**  **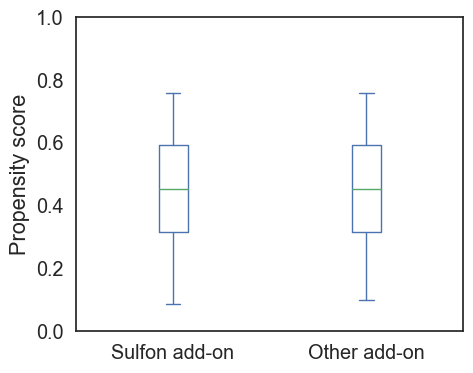** | **(B)**  **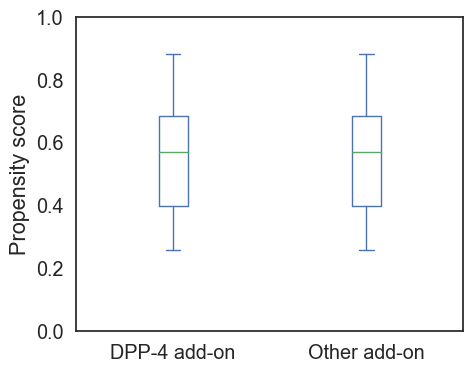** |
| --- | --- |
| **(C)**  **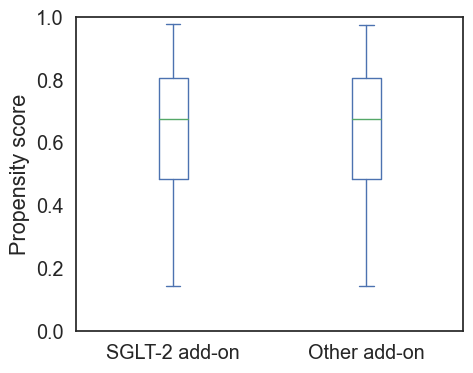** |  |

**Table S5 Univariate analysis – SU add-on**

|  | **HbA_1c_ reduction ≥1% measured at 6^th^ month** | | **HbA_1c_ goal attainment <7% measured at 12^th^ month** | |
| --- | --- | --- | --- | --- |
| **Variable** | **Effect estimate** | **P-value** | **Effect estimate** | **P-value** |
| Age | -0.075 | 0.229 | 0.187 | **0.003** |
| Sex (1 Male 0 Female) | 0.052 | 0.400 | 0.082 | 0.181 |
| Race: (Ref: Chinese) |  |  |  |  |
| Indian | 0.288 | 0.142 | -0.293 | 0.141 |
| Malay | 0.326 | **0.057** | -0.317 | 0.069 |
| Others | -0.321 | 0.408 | -0.459 | 0.226 |
| Body mass index (BMI) | 0.099 | 0.111 | -0.114 | 0.069 |
| Disease duration |  |  |  |  |
| Diabetes (DM) years | 0.013 | 0.830 | 0.177 | **0.004** |
| Hyperlipidaemia (HLD) years | 0.081 | 0.192 | 0.213 | **0.001** |
| Hypertension (HTN) years | 0.148 | **0.017** | 0.260 | **0.000** |
| Lab test results |  |  |  |  |
| Blood pressure systolic (SBP), mmHg | 0.146 | **0.019** | 0.019 | 0.760 |
| Blood pressure diastolic (DBP), mmHg | 0.111 | 0.074 | -0.043 | 0.489 |
| Glycated haemoglobin (HbA1c), % (continuous) | 1.275 | **0.000** | -0.588 | **0.000** |
| Cholesterol high-density lipoprotein (HDL), mmol/L | -0.064 | 0.306 | 0.198 | **0.001** |
| Cholesterol low-density lipoprotein (LDL), mmol/L | 0.035 | 0.569 | -0.269 | **0.000** |
| Triglycerides, mmol/L | 0.054 | 0.383 | -0.226 | **0.000** |
| estimated glomerular filtration rate (eGFR) | -0.044 | 0.478 | -0.187 | **0.003** |
| Creatinine serum | 0.050 | 0.421 | 0.160 | **0.010** |
| Potassium serum | -0.011 | 0.863 | -0.075 | 0.224 |
| Alanine transaminase (ALT) serum | 0.101 | 0.106 | -0.159 | **0.011** |
| Metformin total daily dose (Ref: Low TDS <=1,000mg) |  |  |  |  |
| TDS 1,000mg to >= 2,000mg | 0.028 | 0.852 | 0.148 | 0.323 |
| TDS > 2,000mg | -0.425 | **0.017** | 0.021 | 0.905 |
| Number of HLD medications | 0.079 | 0.201 | 0.005 | 0.934 |
| Number of HTN medications | 0.020 | 0.747 | 0.159 | **0.010** |
| Existing HLD and HTN medications |  |  |  |  |
| HMG-CoA reductase inhibitors | -0.001 | 0.992 | 0.076 | 0.226 |
| Fibric acid derivatives | 0.046 | 0.453 | -0.007 | 0.913 |
| Other lipid-lowering medications | 0.057 | 0.376 | 0.052 | 0.425 |
| Diuretics | 0.073 | 0.232 | 0.110 | 0.073 |
| Beta blockers | 0.032 | 0.611 | 0.075 | 0.220 |
| ACE inhibitors | 0.047 | 0.453 | 0.020 | 0.750 |
| Angiotensin-II receptor | -0.145 | **0.022** | 0.023 | 0.712 |
| Calcium antagonists | 0.050 | 0.423 | 0.205 | **0.001** |
| Other BP-lowering medications | -0.044 | 0.506 | 0.067 | 0.284 |
| Complications |  |  |  |  |
| Macrovascular | -0.014 | 0.827 | -0.058 | 0.349 |
| Renal | 0.035 | 0.576 | 0.078 | 0.202 |
| Eye | 0.052 | 0.405 | 0.092 | 0.136 |
| Foot | -0.011 | 0.858 | -0.012 | 0.842 |

Bold values indicate significant p-value of <0.05.

**Table S6 Univariate analysis – DPP-4 add-on**

|  | **HbA_1c_ reduction ≥1% measured at 6^th^ month** | | **HbA_1c_ goal attainment <7% measured at 12^th^ month** | |
| --- | --- | --- | --- | --- |
| **Variable** | **Effect estimate** | **P-value** | **Effect estimate** | **P-value** |
| Age | -0.064 | 0.482 | 0.285 | **0.002** |
| Sex (1 Male 0 Female) | 0.073 | 0.421 | 0.089 | 0.314 |
| Race: (Ref: Chinese) |  |  |  |  |
| Indian | -0.269 | 0.354 | 0.074 | 0.784 |
| Malay | 0.376 | 0.171 | -0.371 | 0.181 |
| Others | 0.446 | 0.216 | 0.074 | 0.837 |
| Body mass index (BMI) | 0.156 | 0.085 | -0.238 | **0.009** |
| Disease duration |  |  |  |  |
| Diabetes (DM) years | -0.112 | 0.219 | 0.142 | 0.109 |
| Hyperlipidaemia (HLD) years | 0.001 | 0.995 | 0.182 | **0.040** |
| Hypertension (HTN) years | 0.155 | 0.090 | 0.227 | **0.011** |
| Lab test results |  |  |  |  |
| Blood pressure systolic (SBP), mmHg | 0.113 | 0.211 | -0.036 | 0.679 |
| Blood pressure diastolic (DBP), mmHg | 0.133 | 0.145 | -0.133 | 0.134 |
| Glycated haemoglobin (HbA1c), % (continuous) | 1.558 | **0.000** | -0.742 | **0.000** |
| Cholesterol high-density lipoprotein (HDL), mmol/L | -0.150 | 0.108 | -0.008 | 0.931 |
| Cholesterol low-density lipoprotein (LDL), mmol/L | 0.204 | **0.025** | -0.240 | **0.010** |
| Triglycerides, mmol/L | -0.058 | 0.523 | -0.054 | 0.538 |
| estimated glomerular filtration rate (eGFR) | 0.007 | 0.940 | -0.400 | **0.000** |
| Creatinine serum | 0.010 | 0.912 | 0.296 | **0.001** |
| Potassium serum | -0.149 | 0.109 | -0.036 | 0.687 |
| Alanine transaminase (ALT) serum | 0.123 | 0.178 | -0.172 | **0.054** |
| Metformin total daily dose (Ref: Low TDS <=1,000mg) |  |  |  |  |
| TDS 1,000mg to >= 2,000mg | -0.219 | 0.301 | -0.136 | 0.500 |
| TDS > 2,000mg | 0.427 | 0.076 | -0.204 | 0.391 |
| Number of HLD medications | -0.210 | **0.047** | 0.107 | 0.229 |
| Number of HTN medications | 0.047 | 0.603 | 0.201 | **0.024** |
| Existing HLD and HTN medications |  |  |  |  |
| HMG-CoA reductase inhibitors | 0.063 | 0.496 | 0.011 | 0.905 |
| Fibric acid derivatives | -0.220 | **0.033** | 0.114 | 0.199 |
| Other lipid-lowering medications | -0.287 | 0.069 | 0.263 | **0.025** |
| Diuretics | 0.006 | 0.948 | 0.046 | 0.601 |
| Beta blockers | 0.104 | 0.247 | 0.095 | 0.278 |
| ACE inhibitors | 0.065 | 0.472 | 0.014 | 0.875 |
| Angiotensin-II receptor | -0.029 | 0.752 | 0.245 | **0.006** |
| Calcium antagonists | 0.044 | 0.630 | 0.134 | 0.128 |
| Other BP-lowering medications | 0.046 | 0.603 | 0.106 | 0.295 |
| Complications |  |  |  |  |
| Macrovascular | 0.135 | 0.131 | 0.086 | 0.330 |
| Renal | 0.038 | 0.673 | 0.243 | **0.006** |
| Eye | 0.151 | 0.095 | 0.091 | 0.299 |
| Foot | 0.048 | 0.591 | 0.159 | 0.092 |

Bold values indicate significant p-value of <0.05.

**Table S7 Univariate analysis – SGLT-2 add-on**

|  | **HbA_1c_ reduction ≥1% measured at 6^th^ month** | | **HbA_1c_ goal attainment <7% measured at 12^th^ month** | |
| --- | --- | --- | --- | --- |
| **Variable** | **Effect estimate** | **P-value** | **Effect estimate** | **P-value** |
| Age | -0.074 | 0.545 | -0.106 | 0.390 |
| Sex (1 Male 0 Female) | 0.147 | 0.226 | 0.051 | 0.675 |
| Race: (Ref: Chinese) |  |  |  |  |
| Indian | 0.209 | 0.570 | -0.567 | 0.121 |
| Malay | 0.100 | 0.778 | -0.232 | 0.512 |
| Others | -0.370 | 0.543 | -0.090 | 0.876 |
| Body mass index (BMI) | -0.012 | 0.920 | 0.020 | 0.872 |
| Disease duration |  |  |  |  |
| Diabetes (DM) years | 0.003 | 0.977 | -0.046 | 0.704 |
| Hyperlipidaemia (HLD) years | -0.124 | 0.302 | -0.135 | 0.279 |
| Hypertension (HTN) years | 0.100 | 0.417 | -0.092 | 0.458 |
| Lab test results |  |  |  |  |
| Blood pressure systolic (SBP), mmHg | 0.175 | 0.154 | 0.026 | 0.834 |
| Blood pressure diastolic (DBP), mmHg | 0.307 | **0.014** | -0.050 | 0.684 |
| Glycated haemoglobin (HbA_1c_), % (continuous) | 2.163 | **0.000** | -0.362 | **0.004** |
| Cholesterol high-density lipoprotein (HDL), mmol/L | -0.335 | **0.012** | 0.017 | 0.890 |
| Cholesterol low-density lipoprotein (LDL), mmol/L | 0.156 | 0.198 | 0.087 | 0.482 |
| Triglycerides, mmol/L | 0.076 | 0.533 | -0.036 | 0.770 |
| estimated glomerular filtration rate (eGFR) | 0.011 | 0.925 | -0.203 | 0.103 |
| Creatinine serum | 0.094 | 0.442 | 0.276 | **0.029** |
| Potassium serum | -0.065 | 0.595 | -0.097 | 0.429 |
| Alanine transaminase (ALT) serum | -0.024 | 0.846 | 0.175 | 0.160 |
| Metformin total daily dose (Ref: Low TDS <=1,000mg) |  |  |  |  |
| TDS 1,000mg to >= 2,000mg | 0.316 | 0.245 | -0.390 | 0.152 |
| TDS > 2,000mg | 0.051 | 0.881 | 0.149 | 0.672 |
| Number of HLD medications | 0.107 | 0.369 | 0.057 | 0.651 |
| Number of HTN medications | 0.043 | 0.723 | -0.163 | 0.179 |
| Existing HLD and HTN medications |  |  |  |  |
| HMG-CoA reductase inhibitors | -0.060 | 0.614 | -0.062 | 0.619 |
| Fibric acid derivatives | 0.152 | 0.199 | 0.077 | 0.542 |
| Other lipid-lowering medications | -0.025 | 0.842 | -0.082 | 0.483 |
| Diuretics | 0.157 | 0.182 | -0.069 | 0.562 |
| Beta blockers | 0.095 | 0.430 | -0.252 | **0.036** |
| ACE inhibitors | -0.250 | 0.059 | -0.097 | 0.417 |
| Angiotensin-II receptor | 0.148 | 0.221 | -0.033 | 0.786 |
| Calcium antagonists | 0.133 | 0.273 | 0.011 | 0.928 |
| Other BP-lowering medications | 1.576 | 1.000 | 1.512 | 1.000 |
| Complications |  |  |  |  |
| Macrovascular | -0.162 | 0.202 | -0.129 | 0.280 |
| Renal | -0.151 | 0.254 | -0.245 | **0.040** |
| Eye | -0.209 | 0.091 | -0.132 | 0.281 |
| Foot | -0.130 | 0.354 | 0.122 | 0.384 |

Bold values indicate significant p-value of <0.05.

**Text S2** Supplementary criterion HbA_1c_ reduction of >0.5% at the 6-month point.

In our study, we observed that the number and proportion of the patients who achieved a HbA_1c_ reduction >0.5% at 6 months were 674(62.3%), 346(65.4%) and 203(70.0%) for SU, DPP-4 and SGLT-2 add-on medications respectively. While number and proportion of the patients on SU, DPP-4 and SGLT-2 add-on medications achieve HbA_1c_ reduction >1% at 6 months were 436(40.3%), 195(37.3%) and 108(37.2%) respectively.

These observations were in line with the previous studies that compares efficiency of the three add-on medications in lowering HbA_1c_ for patients on metformin therapy. Studies showed that SUs tend to be more effective than DPP-4 in reducing HbA_1c_ over the short term (i.e. reduction at 2% in 3 months), but such differences are mostly lost in the long run (i.e. 1 y­ear) ^1,2^. A study comparing the efficiency of DPP-4 vs SGLT-2 found SGLT-2 to be more efficient than DPP-4 in lowering HbA_1c_ but this difference vanished when the mean baseline HbA_1c_ was ≥ 8% ^3^.

**Univariate Analysis**

For the supplementary criterion on HbA_1c_ reduction of >0.5%, the significant factors for SU add-on medications (i.e. baseline HbA1c, disease duration of DM, HLD and HTN, number of HLD and HTN medications, and calcium antagonist medications class) largely overlaps with the two primary outcomes of HbA_1c_ reduction and goal attainment respectively. For DPP-4 add-on medication, similar to HbA_1c_ reduction >1%, most variables appeared insignificant except baseline HbA_1c_, cholesterol LDL, and ALT serum. For SGLT-2 add-on medication, the significant factors associated with HbA1c reduction of >0.5% at 6 month are baseline HbA_1c_ cholesterol HDL and renal complications.”

**Multivariate Analysis**

For the supplementary criterion on HbA_1c_ reduction of >0.5% at the 6 month time point, patient variables associated with treatment response for SU, DDP-4 and SGLT-2 add-on medications are shown in Supplementary Figure 2. Higher baseline HbA_1c_, an increased number of HTN medications, and the calcium antagonist medication class were independently associated with an increased likelihood of achieving an HbA_1c_ reduction of >0.5% at 6 months for SU add-on medications. Meanwhile, higher HbA_1c_, a longer duration of HLD years, and higher levels of LDL cholesterol were independently associated with an increased likelihood of achieving an HbA_1c_ reduction of >0.5% in 6 months for DPP-4 add-on medications. Higher HbA_1c_, lower HDL cholesterol levels, and the absence of renal complications were associated with a higher likelihood of achieving an HbA_1c_ reduction of >0.5% in 6 months for SGLT-2 add-on medications.

**Figure S2** Multivariable analysis for all significant variables for Supplementary criterion HbA_1c_ reduction of >0.5% at the 6-month point

| **(A)** Multivariable analysis on supplementary criterion - HbA_1c_ reduction of >0.5% at 6 month for SU add-on medication  **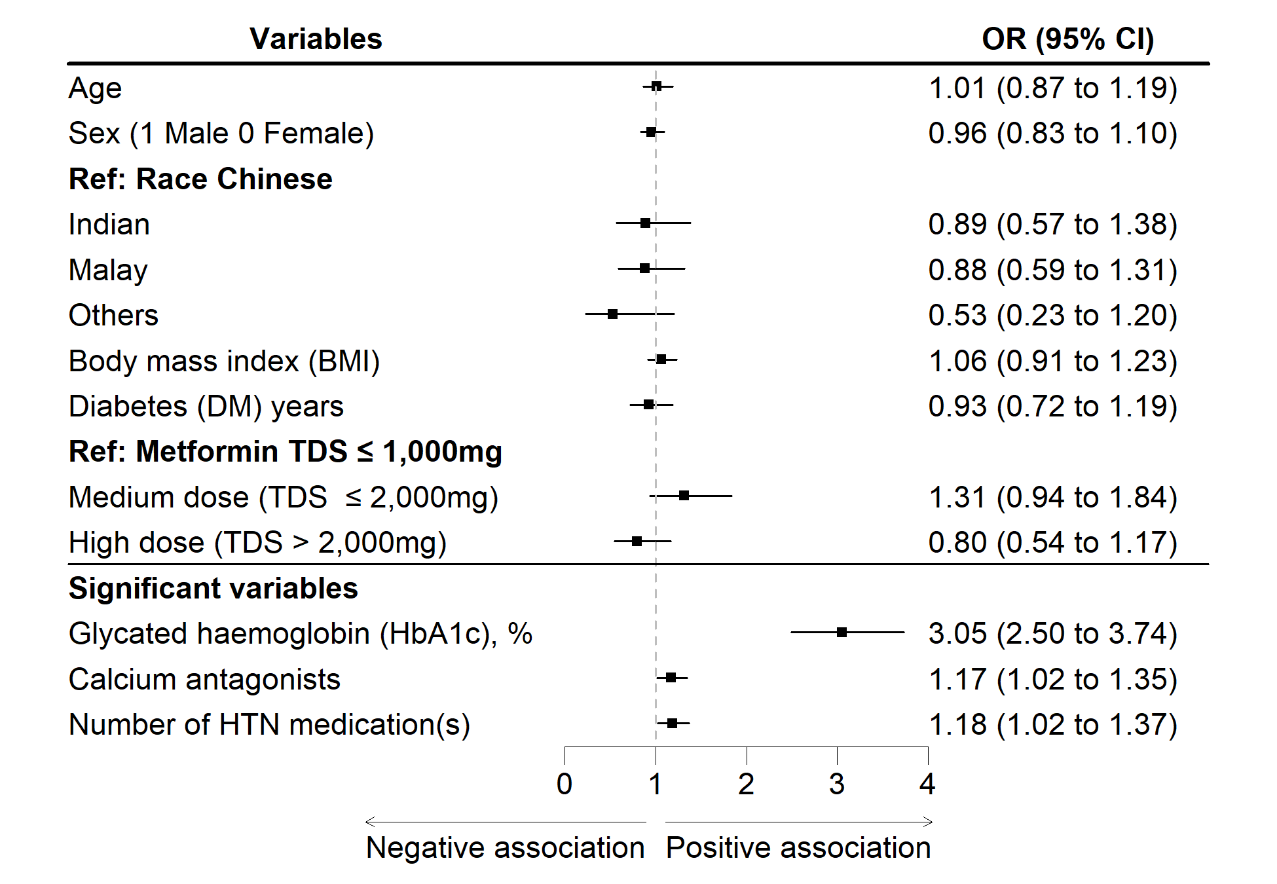** |
| --- |
| **(B)** Multivariable analysis on supplementary criterion - HbA_1c_ reduction of >0.5% at 6 month for DPP-4 add-on medication  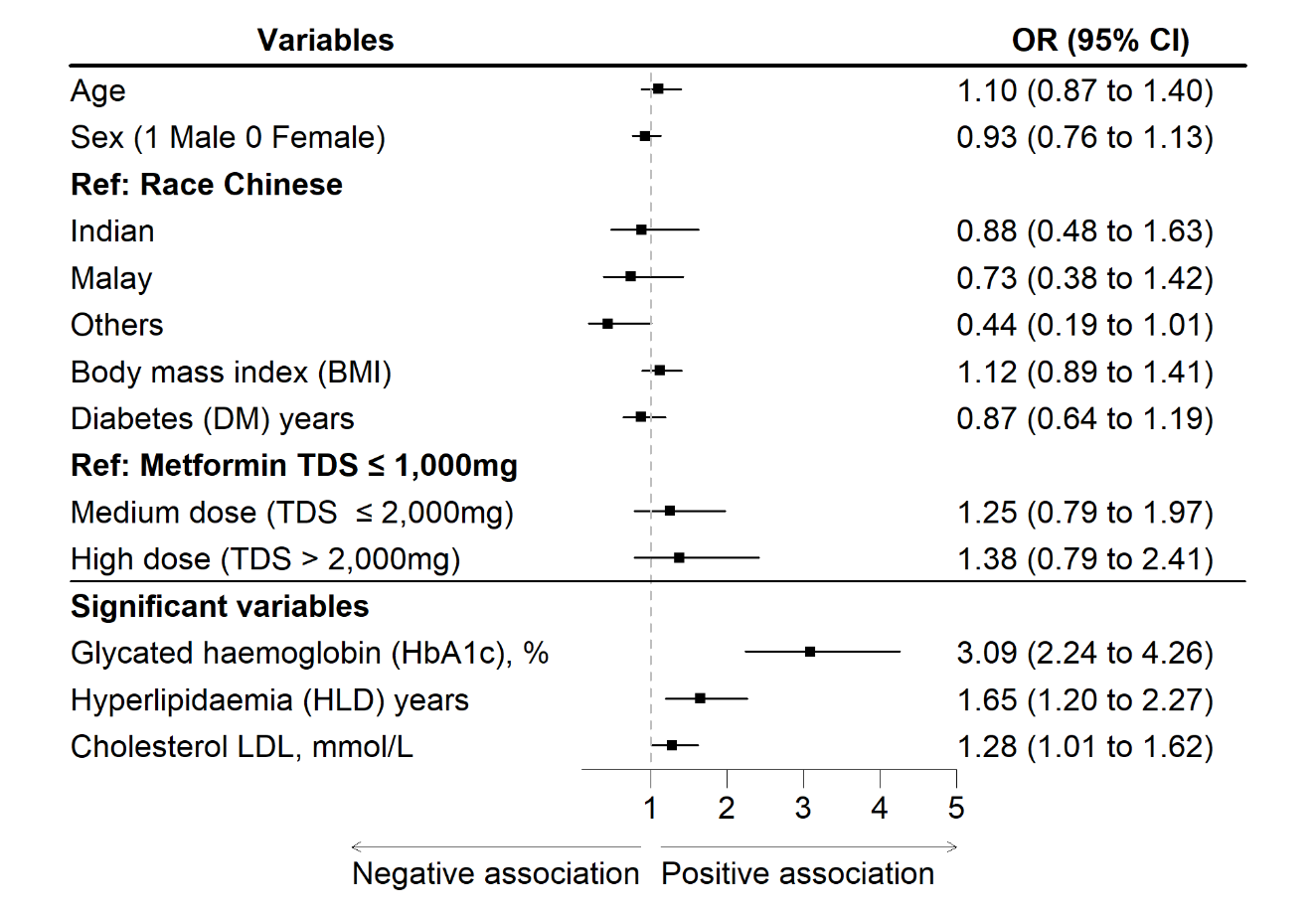 |
| **(C)** Multivariable analysis on supplementary criterion - HbA_1c_ reduction of >0.5% at 6 month for SGLT-2 add-on medication  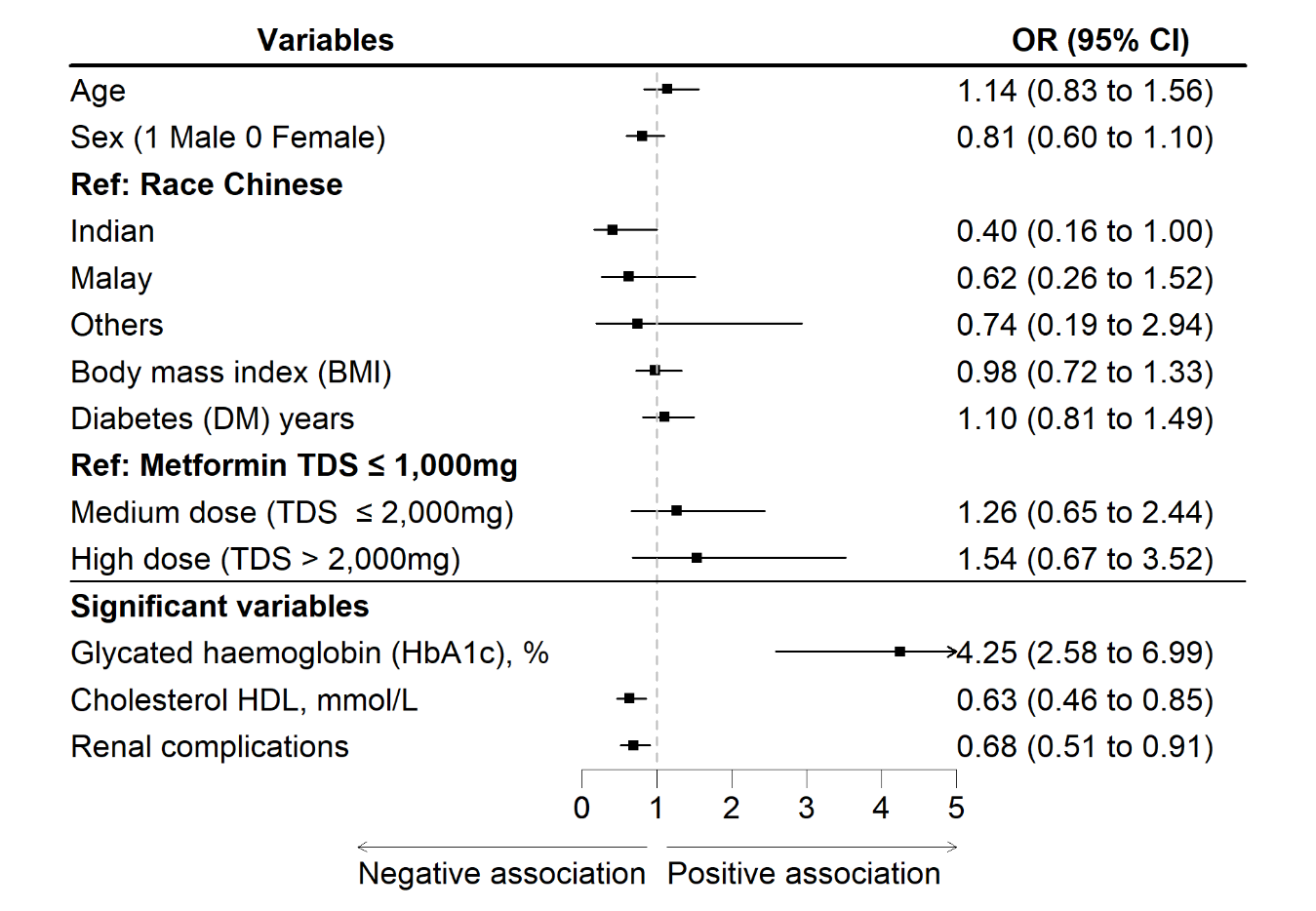 |

Column in right indicates odds ratio and its 95% confidence interval (CI) in brackets. Black box represents the odds ratio, and the horizontal line represents the 95%CI. Dotted vertical indicates line of null effect. All models were adjusted for age, sex, race, BMI, disease duration of diabetes and baseline metformin total daily dose.

**Table S8 Multivariable analysis odds ratio and 95%CI for all significant variables for HbA1c reduction at 6^th^ month**

|  | **SU (n=1,073)** | | **DPP-4 (n=517)** | | **SGLT-2 (n=290)** | |
| --- | --- | --- | --- | --- | --- | --- |
| **Variables** | **OR** | **95%CI** | **OR** | **95%CI** | **OR** | **95%CI** |
| Age | 0.93 | (0.79,1.09) | 1.12 | (0.88,1.42) | 0.91 | (0.65,1.27) |
| Sex (1 Male 0 Female) | 0.99 | (0.86,1.15) | 1.15 | (0.93,1.42) | 1.07 | (0.78,1.48) |
| Race Chinese reference |  |  |  |  |  |  |
| Malay | 1.22 | (0.78,1.93) | 0.64 | (0.32,1.28) | 0.87 | (0.32,2.37) |
| Indian | 1.07 | (0.72,1.6) | 1.16 | (0.6,2.24) | 0.86 | (0.32,2.27) |
| Others | 0.49 | (0.18,1.31) | 1.11 | (0.48,2.6) | 0.39 | (0.08,1.82) |
| Body mass index (BMI) | 1.02 | (0.87,1.19) | 1.05 | (0.83,1.33) | 0.93 | (0.67,1.3) |
| Diabetes (DM) years | 0.84 | (0.68,1.04) | 1.06 | (0.85,1.33) | 1.02 | (0.74,1.4) |
| Metformin low dose (TDS ≤ 1,000mg) reference |  |  |  |  |  |  |
| Medium dose (TDS ≤ 2,000mg) | 1.02 | (0.72,1.44) | 0.58 | (0.35,0.95) | 1.15 | (0.57,2.35) |
| High dose (TDS > 2,000mg) | 0.75 | (0.5,1.13) | 1.18 | (0.68,2.05) | 0.97 | (0.39,2.39) |
| **Significant Variables** |  |  |  |  |  |  |
| Glycated haemoglobin (HbA1c), % (continuous) | 3.70 | (3.05,4.5) | 5.03 | (3.56,7.12) | 9.20 | (5.2,16.28) |
| Hypertension (HTN) years | 1.56 | (1.26,1.94) |  |  |  |  |
| Cholesterol high-density lipoprotein (HDL), mmol/L |  |  |  |  | 0.70 | (0.5,0.98) |

The multivariate linear regression model was adjusted known covariates of HbA1c response (i.e. age, sex, ethnicity, BMI, diabetes duration and baseline metformin total daily dose). SU: Sulfonylureas; DPP-4 inhibitor: Dipeptidyl peptidase-4 inhibitor; SGLT-2 inhibitor: Sodium-glucose cotransporter-2 inhibitors

**Table S9 Multivariable analysis odds ratio and 95%CI for all significant variables for HbA_1c_ goal attainment at 12^th^ month**

|  | **SU (n=1,073)** | | **DPP-4 (n=517)** | | **SGLT-2 (n=290)** | |
| --- | --- | --- | --- | --- | --- | --- |
| **Variables** | **OR** | **95%CI** | **OR** | **95%CI** | **OR** | **95%CI** |
| Age | 1.02 | (0.88,1.18) | 1 | (0.78,1.28) | 0.93 | (0.7,1.24) |
| Sex (1 Male 0 Female) | 1.19 | (1.04,1.36) | 1.11 | (0.92,1.34) | 0.79 | (0.58,1.08) |
| Race Chinese reference |  |  |  |  |  |  |
| Malay | 1.01 | (0.66,1.54) | 1.34 | (0.75,2.41) | 0.48 | (0.21,1.06) |
| Indian | 0.9 | (0.62,1.31) | 0.98 | (0.53,1.81) | 0.64 | (0.29,1.41) |
| Others | 0.83 | (0.37,1.82) | 1.28 | (0.59,2.76) | 0.67 | (0.2,2.27) |
| Body mass index (BMI) | 0.92 | (0.8,1.06) | 0.81 | (0.66,1.01) | 1.15 | (0.87,1.52) |
| Diabetes (DM) years | 0.95 | (0.79,1.15) | 1 | (0.82,1.21) | 1.14 | (0.86,1.51) |
| Metformin low dose (TDS ≤ 1,000mg) reference |  |  |  |  |  |  |
| Medium dose (TDS ≤ 2,000mg) | 1.16 | (0.85,1.59) | 1.08 | (0.7,1.66) | 0.81 | (0.45,1.45) |
| High dose (TDS > 2,000mg) | 1.02 | (0.71,1.46) | 1.18 | (0.71,1.98) | 1.56 | (0.73,3.33) |
| **Significant Variables** |  |  |  |  |  |  |
| Glycated haemoglobin (HbA1c), % (continuous) | 0.57 | (0.49,0.66) | 0.51 | (0.4,0.65) | 0.68 | (0.52,0.88) |
| Hypertension (HTN) years | 1.26 | (1.03,1.53) |  |  |  |  |
| Cholesterol high-density lipoprotein (HDL), mmol/L | 1.24 | (1.08,1.42) |  |  |  |  |
| Creatinine serum |  |  |  |  | 1.82 | (1.28,2.57) |
| estimated glomerular filtration rate (eGFR) |  |  | 0.71 | (0.57,0.9) |  |  |
| Renal complications |  |  |  |  | 0.65 | (0.49,0.86) |
| Beta blockers |  |  |  |  | 0.74 | (0.57,0.97) |
| Angiotensin-II receptor |  |  | 1.26 | (1.04,1.52) |  |  |
| Calcium antagonists | 1.16 | (1.01,1.33) |  |  |  |  |

The multivariate linear regression model was adjusted known covariates of HbA1c response (i.e. age, sex, ethnicity, BMI, diabetes duration and baseline metformin total daily dose). SU: Sulfonylureas; DPP-4 inhibitor: Dipeptidyl peptidase-4 inhibitor; SGLT-2 inhibitor: Sodium-glucose cotransporter-2 inhibitor.

**Fig S3** C-statistics for HbA_1c_ reduction and HbA1c goal attainment

| **A**  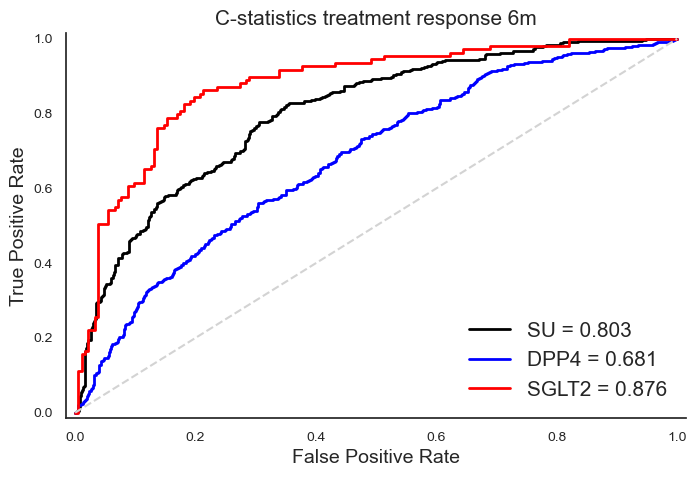 | **B**  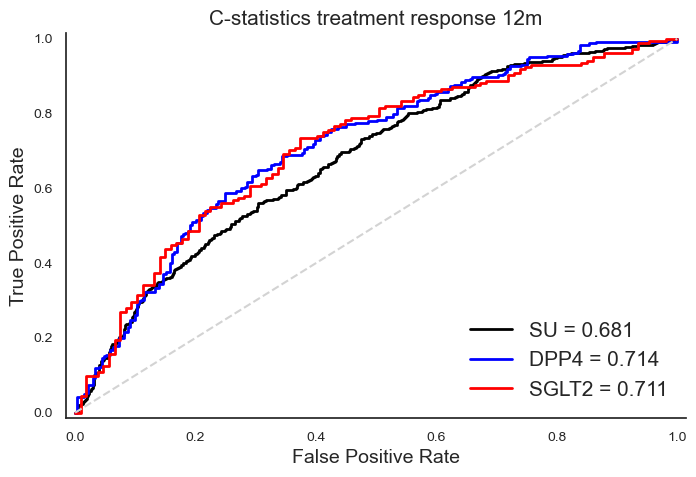 |
| --- | --- |

SU: Sulfonylureas; DPP-4 inhibitor: Dipeptidyl peptidase-4 inhibitor; SGLT-2 inhibitor: Sodium glucose cotransporter-2 inhibitor.

**References**

1 Fadini, G. P. *et al.* Comparative effectiveness of DPP-4 inhibitors versus sulfonylurea for the treatment of type 2 diabetes in routine clinical practice: a retrospective multicenter real-world study. *Diabetes Ther.* **9**, 1477-1490. <https://doi.org:10.1007/s13300-018-0452-y> (2018).

2 Mishriky, B. M., Cummings, D. M. & Tanenberg, R. J. The efficacy and safety of DPP4 inhibitors compared to sulfonylureas as add-on therapy to metformin in patients with type 2 diabetes: a systematic review and meta-analysis. *Diabetes Res. Clin. Pract.* **109**, 378-388. <https://doi.org:10.1016/j.diabres.2015.05.025> (2015).

3 Scheen, A. Reduction in HbA1c with SGLT2 inhibitors vs. DPP-4 inhibitors as add-ons to metformin monotherapy according to baseline HbA1c: a systematic review of randomized controlled trials. *Diabetes Metab.* **46**, 186-196. <https://doi.org:10.1016/j.diabet.2020.01.002> (2020).
